# One new species of *Drapetisca* Menge, 1866 (Araneae, Linyphiidae) from Gele Mountain, Chongqing, China

**DOI:** 10.3897/BDJ.14.e180901

**Published:** 2026-01-13

**Authors:** He-Xiong Shi, Zu-Ying Liu, Muhammad Irfan, Lu-Yu Wang

**Affiliations:** 1 Chongqing Academy of Forestry, Chongqing Urban Ecosystem Observation and Research Station, National Forestry and Grassland Administration, Chongqing, China Chongqing Academy of Forestry, Chongqing Urban Ecosystem Observation and Research Station, National Forestry and Grassland Administration Chongqing China; 2 Chongqing Zhonglinfeng Technology Development Co., Ltd., Chongqing, China Chongqing Zhonglinfeng Technology Development Co., Ltd. Chongqing China; 3 Key Laboratory of Eco-environments in Three Gorges Reservoir Region (Ministry of Education), School of Life Sciences, Southwest University, Chongqing, China Key Laboratory of Eco-environments in Three Gorges Reservoir Region (Ministry of Education), School of Life Sciences, Southwest University Chongqing China; 4 College of Plant Protection, Southwest University, Chongqing, China College of Plant Protection, Southwest University Chongqing China

**Keywords:** description, distribution, morphology, taxonomy, sheet-web spiders

## Abstract

**Background:**

The genus *Drapetisca* comprises 7 species distributed in Canada, Central Asia, China, Caucasus, Europe, Japan, Russia (Europe to the Far East), and the USA, out of which five species have been recorded in China (World Spider Catalog 2025). The males and females of *Drapetisca* can be distinguished from all other Linyphiidae genera by the palpal distal arm of the paracymbium anchor-shaped in the retrolateral view and the protruding epigyne without a scape.

**New information:**

While examining Linyphiidae Blackwall, 1859 specimens from Gele Mountain in Chongqing, we discovered a new species of the genus *Drapetisca*, *D. dentata* **sp. nov.** Detailed descriptions, photographs of copulatory organs and somatic features, and a distribution map are provided.

## Introduction

The Linyphiidae is the second-largest spider family globally, comprising 640 extant genera and 4,963 species, with an additional 11 fossil genera and 62 species (World Spider Catalog 2025). In China, approximately 633 species belonging to 182 genera have been documented (Tanasevitch 2025). The genus *Drapetisca* currently comprises 7 species, of which 5 have been reported from China (World Spider Catalog 2025). While examining the specimens collected from Gele Mountain, Chongqing, a new species of the genus *Drapetisca* was identified and described here.

## Materials and methods

Specimens were collected by handpicking and the sieving leaf litter method, and were preserved in 75% ethanol. The left male palp was used for photography. After dissection, epigynes were cleared in trypsin enzyme solution before examination and photography. The specimens were examined and measured using Leica M205A stereomicroscope equipped with a Leica DFC450 camera and LAS (v. 4.6 software). All the photos of habitus and copulatory organs were taken with a Kuy Nice CCD mounted on an Olympus BX53 compound microscope. Compound focus images were generated using Helicon Focus v. 6.7.1. Eye sizes were measured at the maximum dorsal diameter. Leg measurements are shown as total length (femur, patella, tibia, metatarsus, tarsus). All measurements are in millimeters. Specimens are deposited in the School of Life Sciences, Southwest University, Chongqing (SWUC), China. The map was created using the online mapping software SimpleMappr (Shorthouse 2010). The terminology used in the text and figure legends follows Irfan et al. (2025b).

The following abbreviations are used in the text: AER—anterior eye row; ALE—anterior lateral eyes; AME—anterior median eyes; AME–ALE—the distance between AME and ALE; AME–AME—the distance between AMEs; PER—posterior eye row; PLE—posterior lateral eyes; PME—posterior median eyes; PME–PLE—distance between PME and PLE; PME–PME—distance between PMEs.

## Taxon treatments

### Drapetisca dentata sp. nov.

92B8A3E0-1017-5C5C-98AE-C1CB9330D9B5

9A3EF86E-1B2B-4788-8663-159B01F884AC

#### Materials

- **Type status:** Holotype. **Occurrence:** recordedBy: Luyu Wang, Muhammad Irfan, Muyan Nan, Xiangyun Zhang; individualCount: 1; sex: Male; lifeStage: adult; occurrenceID: 0E944935-E5F5-5148-B35F-3713A5A73C53; **Taxon:** class: Arachnida; order: Araneae; family: Linyphiidae; genus: Drapetisca; **Location:** continent: Asia; country: China; stateProvince: Chongqing; county: Shapingba District; locality: Gele Mountain National Forest Park; verbatimElevation: 590 m; verbatimLatitude: 29°34′17.0520"N; verbatimLongitude: 106°25′20.1960″E; **Event:** samplingProtocol: handpicking and sieving leaf litter; year: 2025; month: 1; day: 5; habitat: subtropical evergreen broad-leaved forests; **Record Level:** institutionID: SWUC-T-LIN-41-01
- **Type status:** Paratype. **Occurrence:** recordedBy: Luyu Wang, Muhammad Irfan, Muyan Nan, Xiangyun Zhang; sex: 1 Male, 3 females; lifeStage: adult; occurrenceID: 5279D48B-D4ED-5B17-AFE7-E24F79004BE8; **Taxon:** class: Arachnida; order: Araneae; family: Linyphiidae; genus: Drapetisca; **Location:** continent: Asia; country: China; stateProvince: Chongqing; county: Shapingba District; locality: Gele Mountain National Forest Park; verbatimElevation: 590 m; verbatimLatitude: 29°34′17.0520"N; verbatimLongitude: 106°25′20.1960″E; **Event:** samplingProtocol: handpicking and sieving leaf litter; year: 2025; month: 1; day: 5; habitat: subtropical evergreen broad-leaved forests; **Record Level:** institutionID: SWUC-T-LIN-41-02~05

#### Description

**Figure 1.**
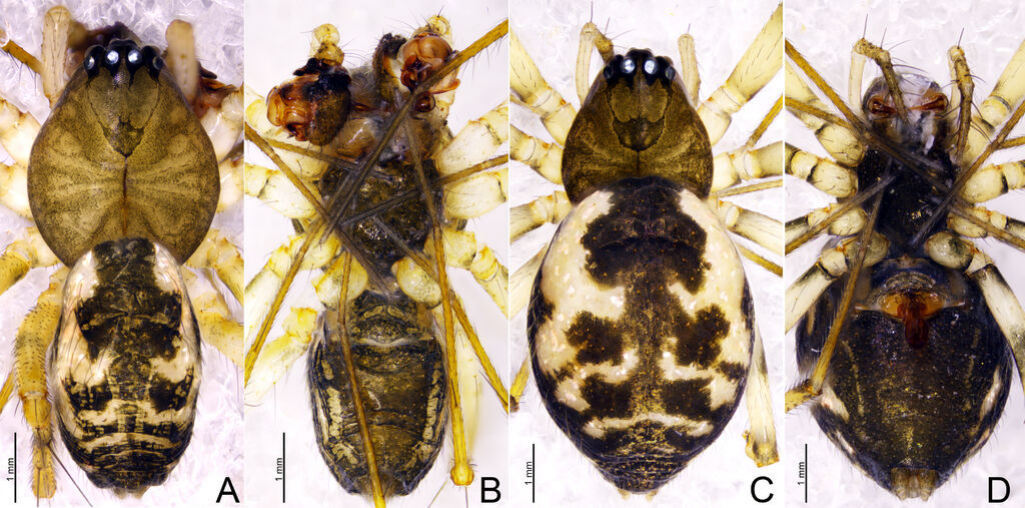
*Drapetisca dentata* sp. nov., male holotype (A, B) and female paratype (C, D). **A**, **C** Habitus, dorsal view **B, D** Habitus, ventral view.

**Male** (holotype, SWUC-T-LIN-41-01, Fig. 1A, B): Total length: 2.38. Carapace 1.13 long, 0.96 wide, yellowish grey, fovea, cervical and radial grooves distinct (Fig. 1A, B). Clypeus 0.24 high. Chelicerae with 1 promarginal and 1 retromarginal tooth. AER recurved, PER slightly recurved and wider. Sternum as long as wide, dark brown, with microsetae. Eye sizes and interdistances: AME 0.06, ALE 0.10, PME 0.08, PLE 0.09, AME–AME 0.04, PME–PME 0.06, AME–ALE, 0.05, PME–PLE 0.06, AME–PME 0.09, ALE–ALE 0.37, PLE–PLE 0.41, ALE–PLE 0.01. Length of legs: I 5.56 (1.49, 0.37, 1.44, 1.45, 0.81), II 4.94 (1.31, 0.34, 1.25, 1.26, 0.78), III 3.69 (1.03, 0.31, 0.87, 0.92, 0.56), IV 5.33 (1.33, 0.28, 1.22, 1.24, 1.26). Tibial spine formula: 2-2-2-2. TmI 0.56 and TmIV present. Opisthosoma 1.42 long, 0.86 wide, oval, dorsally with a distinct black pattern with white patches; ventral side blackish brown (Fig. 1A, B).

**Figure 2.**
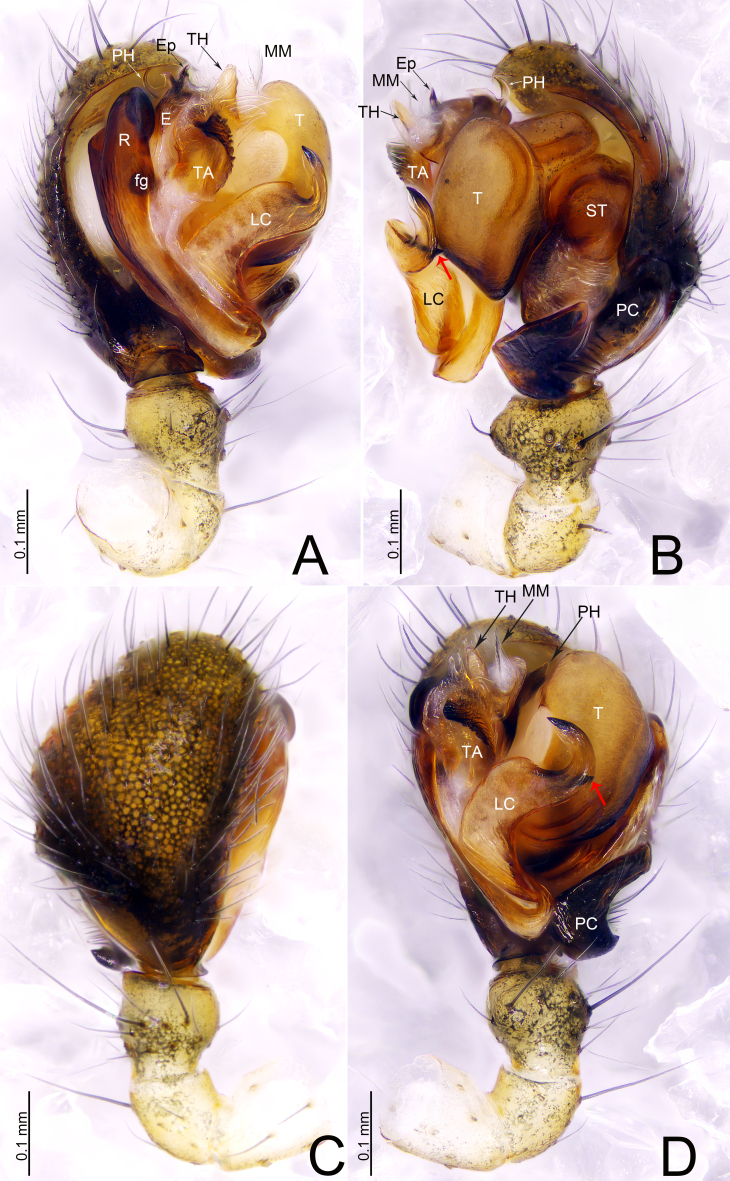
*Drapetisca dentata* sp. nov., male holotype. **A** Palp, prolateral view **B** Palp, retrolateral view **C** Palp, dorsal view **D** Palp, ventral view. Abbreviations: E—embolus; EP—embolus proper; fg—Fickert’s gland; LC—lamella characteristica; MM—median membrane; PC—paracymbium; R—radix; ST—subtegulum; T—tegulum; TA—terminal apophysis; TH—thumb.

**Palp** (Fig. 2A–D). Femur unmodified, 2 times longer than both patella and tibia. Patella shorter than tibia, ventrally grooved, with dorsal spine. Tibia conical, ventrally with outgrowth; with 2 retrolateral and 1 dorsal trichobothria. Cymbium unmodified, retrolateral margin with a shallow depression at the base, retrolateral margin protruding towards subtegulum; paracymbium (PC) basal part wider than long, with thick spines, distal arm with a small projection at the center, tip with blunt end (Fig. 2B). Pithook (PH) bifurcated, apical end membranous. Radix (R) longer than wide with Fickert’s gland (fg) (Fig. 2A); terminal apophysis (TA) sclerotized, apical end curved, ventral surface ridged (Fig. 2A, D); lamella characteristica (LC) longer than wide, tip hook-shaped, apical part enclosed in a membranous sheet, extending forward (Fig. Fig. 2A, B, D); embolus (E) longer than wide, embolus proper (EP) short with pointed tip; attached with hammer-shaped thumb (TH) (Fig. 2A, B, D).

**Female** (paratype, SWUC-T-LIN-41-02, Fig. 1C, D): Total length: 2.49. Carapace 0.98 long, 0.83 wide, cephalic region slightly elevated, yellowish grey, fovea, cervical and radial grooves distinct (Fig. 1C, D). Clypeus 0.19 high. Chelicerae with 4 promarginal and 4 retromarginal teeth. Sternum longer than wide, black with light patches and microsetae. Eyes: AER recurved, PER slightly recurved and wider. Eye sizes and interdistances: AME 0.05, ALE 0.09, PME 0.07, PLE 0.09, AME–AME 0.03, PME–PME 0.07, AME–ALE, 0.05, PME–PLE 0.06, AME–PME 0.10, ALE–ALE 0.37, PLE–PLE 0.39, ALE–PLE 0.01. Legs with annuli. Length of legs: I 4.57 (1.23, 0.34, 1.19, 1.08, 0.73), II 4.06 (1.12, 0.32, 0.94, 1.02, 0.66), III 3.19 (0.91, 0.25, 0.71, 0.74, 0.58), IV 4.12 (1.13, 1.23, 1.03, 0.61). Tibial spine formula: 2-2-2-2. TmI 0.58 and TmIV present. Opisthosoma 1.69 long, 1.13 wide, all other characters same as in male, except darker in color (Fig. 1C, D).

**Figure 3.**
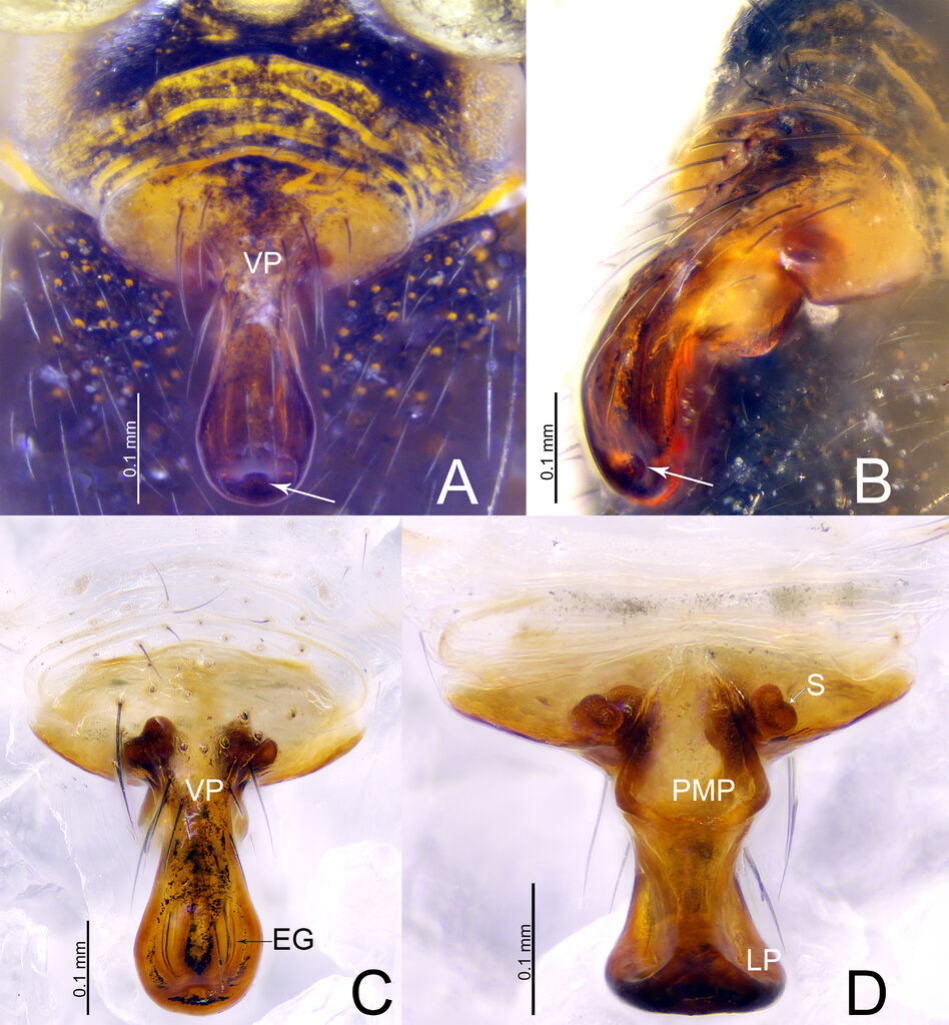
*Drapetisca dentata* sp. nov., female paratype. **A, C** Epigyne, ventral view **B** Epigyne, lateral view **D** Epigyne, dorsal view. Abbreviations: EG —entrance groove; LP—lateral pocket; PMP—posterior median plate; S—spermatheca; VP—ventral plate.

**Epigyne** (Fig. 3A–D). Protruding, ventral plate posteriorly longer than wide, distal end drop-shaped with distinct depression, lateral pockets (LP) indistinct, entrance groove (EG) present laterally (Fig. 3C, D); posterior median plate (PMP) small, somewhat rectangular (Fig. 3D). Spermathecae (S) 9-shaped, present antero-laterally (Fig. 3D).

#### Diagnosis

*Drapetisca dentata* sp. nov. resembles *D. socialis* (Sundevall, 1833) in having the similar paracymbium in male palp and epigyne with similar morphology (Fig. 2A–D; Locket and Millidge 1953, fig. 222A–B, E, H), but can be distinguished by the lamella characteristica hook-shaped, distal end with a tooth in retrolateral view in *D. dentata* sp. nov. (Fig. 2; vs. strongly curved, tip with 4 branches in *D. socialis*); proximal cymbial apophysis absent in both *D. dentata* sp. nov. (Fig. 2C; vs. present in *D. socialis*). The female can be distinguished by the posterior part of the ventral plate drop-shaped, with a depression at the apex in ventral view in *D. dentata* sp. nov. (Fig. 3A–D; vs. somewhat rectangular in *D. socialis*).

#### Etymology

The specific epithet is derived from the Latin adjective “*dentatus*,” meaning “toothed,” and referring to the distal part of the lamella characteristica apically with a tooth.

#### Distribution

**Figure 4.**
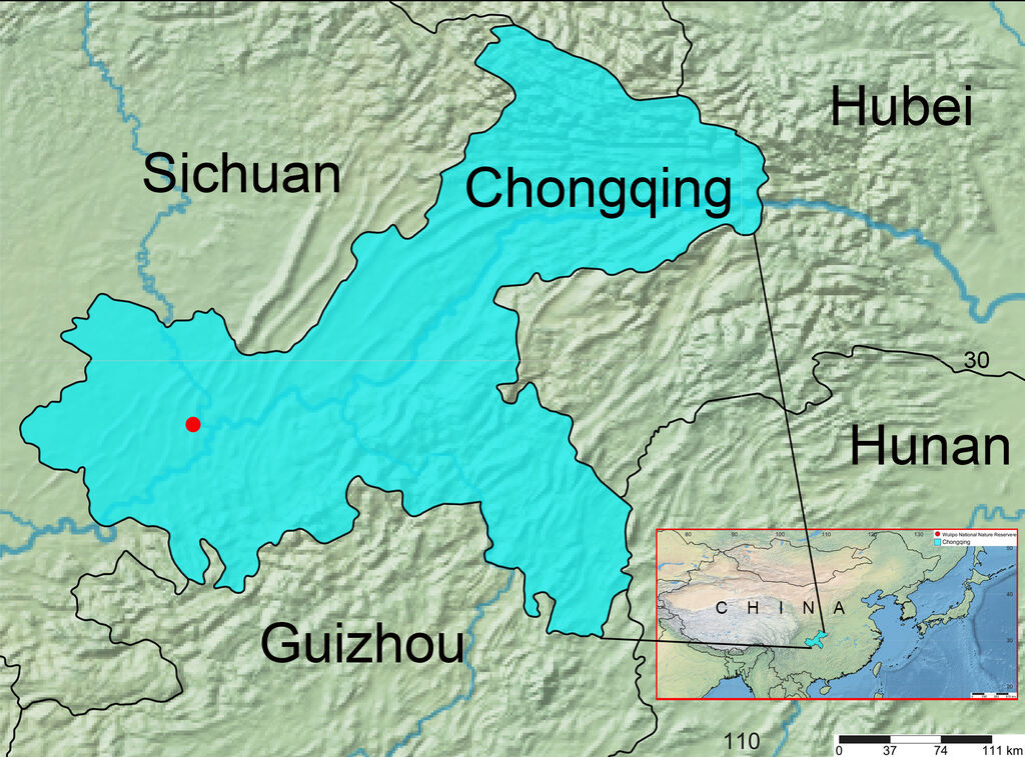
Distribution of *Drapetisca dentata* in Gele Mountain, Chongqing.

Known only from the type locality (Fig. 4).

## Discussion

Over the past four years, a significant increase in the diversity of Linyphiidae spiders from Chongqing has been observed, with approximately 63 species across 40 genera added to the records (Irfan et al. 2022, Irfan et al. 2023a, Irfan et al. 2023b, Irfan et al. 2024, Irfan et al. 2025a, Irfan et al. 2025b). The new species *Drapetisca dentata* **sp. nov.** is known only from the Palearctic part of China, consistent with the distribution pattern observed in all other Drapetisca species recorded from the country, none of which extended to the Oriental Region. The discovery of *Drapetisca dentata* sp. nov. in Gele Mountain National Forest Park, Chongqing, has significant implications for biodiversity research and conservation. First, it fills a gap in the distribution of the genus *Drapetisca*, which was previously unrecorded in Chongqing, despite its presence in neighboring Hunan (Irfan et al. 2025b). This finding underscores the ecological significance of Gele Mountain, a biodiversity hotspot renowned for its rich endemic flora and fauna. Second, it underscores the value of protected areas like Gele Mountain National Forest Park in conserving understudied arthropod groups, emphasizing the need for systematic surveys to uncover hidden species and refine conservation priorities. Lastly, this discovery not only enriches China’s Linyphiidae diversity but also emphasizes the urgency of preserving montane ecosystems amid growing anthropogenic pressures.

## Supplementary Material

XML Treatment for Drapetisca dentata
